# Peristaltic flow in the glymphatic system

**DOI:** 10.1038/s41598-020-77787-4

**Published:** 2020-12-03

**Authors:** Francesco Romanò, Vinod Suresh, Peter A. Galie, James B. Grotberg

**Affiliations:** 1 Univ. Lille, CNRS, ONERA, Arts et Métiers Institute of Technology, Centrale Lille, UMR 9014 - LMFL - Laboratoire de Mécanique des Fluides de Lille - Kampé de Fériet, 59000 Lille, France; 2 Auckland Bioeng. Inst. and Dept. Eng. Sci., University of Auckland, 70 Symonds Street, Bldg 439, Auckland, 1010 New Zealand; 3 Dept. Biomed. Eng., Rowan University, 201 Mullica Hill Rd, Glassboro, NJ 08028 USA; 4 Dept. Biomed. Eng., University of Michigan, 2123 Carl A. Gerstacker Building, 2200 Bonisteel Boulevard, Ann Arbor, MI 48109-2099 USA

**Keywords:** Fluid dynamics, Computational models, Biomedical engineering

## Abstract

The flow inside the perivascular space (PVS) is modeled using a first-principles approach in order to investigate how the cerebrospinal fluid (CSF) enters the brain through a permeable layer of glial cells. Lubrication theory is employed to deal with the flow in the thin annular gap of the perivascular space between an impermeable artery and the brain tissue. The artery has an imposed peristaltic deformation and the deformable brain tissue is modeled by means of an elastic Hooke’s law. The perivascular flow model is solved numerically, discovering that the peristaltic wave induces a steady streaming to/from the brain which strongly depends on the rigidity and the permeability of the brain tissue. A detailed quantification of the through flow across the glial boundary is obtained for a large parameter space of physiologically relevant conditions. The parameters include the elasticity and permeability of the brain, the curvature of the artery, its length and the amplitude of the peristaltic wave. A steady streaming component of the through flow due to the peristaltic wave is characterized by an in-depth physical analysis and the velocity across the glial layer is found to flow from and to the PVS, depending on the elasticity and permeability of the brain. The through CSF flow velocity is quantified to be of the order of micrometers per seconds.

## Introduction

Cerebrospinal fluid serves as a sink for metabolic waste products generated in the brain. The pathway for CSF transport in the brain interstitium has been a puzzle. Recent imaging experiments using in vivo two-photon microscopy have lent support to the hypothesis that CSF enters the brain from the subarachnoid space along the perivascular sheaths surrounding penetrating arteries and ‘leaks’ out into the interstitium across a permeable layer of glial (astrocyte) cells. From there, it is cleared into the perivascular sheaths around veins and the pulsation of the cerebral arteries are identified as an important driver for the transport of CSF into the brain interstitium^1,2^. Since convective bulk flow of the CSF between these ingress and egress pathways facilitates the clearance of solutes and metabolic waste products from brain tissue, dysfunctions in CSF transport may have implication for a range of neurological conditions such as intracranial hypertension and protein clearance in Alzheimer’s disease and Parkinson’s disease. Empirical studies^2–4^ indicate that CSF transport is affected by the elastic properties of vessel walls, water permeability of brain tissue and pulsatility of blood flow. However, the difficulty of measuring these parameters in vivo necessitates modeling-based approaches to improve our understanding of fluid transport in the brain. Therefore, the aim of this study is to develop a mathematical model of perivascular transport that provides insight into how these factors alter the direction and magnitude of CSF flow. Since we aim at deriving a leading-order characterization of the CSF flow, the impact of ciliated boundaries and non-Newtonian effects^5–7^ will be neglected in our model.

The model described here builds upon previous approaches to calculate perivascular fluid flow in idealized geometries. Wang and Olbricht^8^ studied axial flow and transport in an annulus with impermeable boundaries, but did not address fluid exchange with the interstitium^9^. Kyrtsos and Baras modeled protein clearance from the interstitium using a compartmental model in which CSF velocity was an input parameter and was assumed to be inversely proportional to vessel stiffness^10^. Cerebral MRI visualizations of a live rat have been used by Ratner et al.^11^ to find the direction of the glymphatic flow. Moreover, they made use of a purely diffusive model to estimate the liquid flow through the healthy brain of a rat and reproduced the main experimental features by means of an Optimal Mass Transfer approach which could also estimate the diffusion tensor based on the dynamic flow rate. By means of numerical simulations, Asgari et al.^12^ claimed that the arterial pulsation due to the peristaltic wave cannot be the tribological driving force responsible of the interstitial solute transport. They address the role of dispersion transport, which is a combination of local mixing and diffusive effects in the para-arterial space. A very different conclusion has been drawn by Aldea et al.^13^, who proposed a multiscale model of the arteries dealing with the basement membrane as a deformable fluid-filled, poroelastic medium. They rather concluded that the vasomotion-driven intramural periarterial drainage is compatible with experimental observations.

Jin et al.^14^ modeled the glymphatic system from para-arterial to paravenous cerebrospinal fluid through brain extracellular space. They investigated the glymphatic mechanism for solute clearance in brain by modeling diffusive and convective transport in the cerebral extracellular space, focusing on the short-range transport between para-arterial and paravenous spaces. Based on the numerical simulations of their model, they concluded that the convective transport is not affected by the pressure fluctuations and requires a strong pressure gradient to be significant. Moreover, they found that the convective transport is also fairly insensitive to astrocyte endfoot water permeability and that diffusion transport suffices to explain the experimental data of the transport studies in brain parenchyma. Similarly, Faghih and Sharp^15^ use a one-dimensional steady, pressure-driven branching flow model to analyze the hydraulic resistance of arterial membranes. They found that the resistance of the periarterial tree is too great to account for physiological estimates of the CSF leakage rate, and that a combined route through the paraarterial and paravenous spaces would also be unlikely based on the magnitude of the transmantle pressure. A similar approach was employed by Rey and Sarntinoranont^16^, who made use of two resistance network models to study the effect of pulsating flows. They estimated that the peak fluid velocity in the PVS and parenchyma increases with the pulse amplitude and the vessel size, making the convective solute transport less and less relevant.

Our model derives a leading order approximation of the Navier–Stokes equation which is based on lubrication theory and includes the effect of a peristaltic wave in the artery and the deformability of the brain tissue. A further justification of the negligible importance of convective transport compared to diffusion effects in the PVS will be derived from first principles, motivating such a conclusion by dimensional analysis considerations. Thereafter, focusing on CSF exchange between the perivascular space and brain interstitium, we compute the leak velocity using our first-principles approach.

## Model

### Geometry

**Figure 1:**
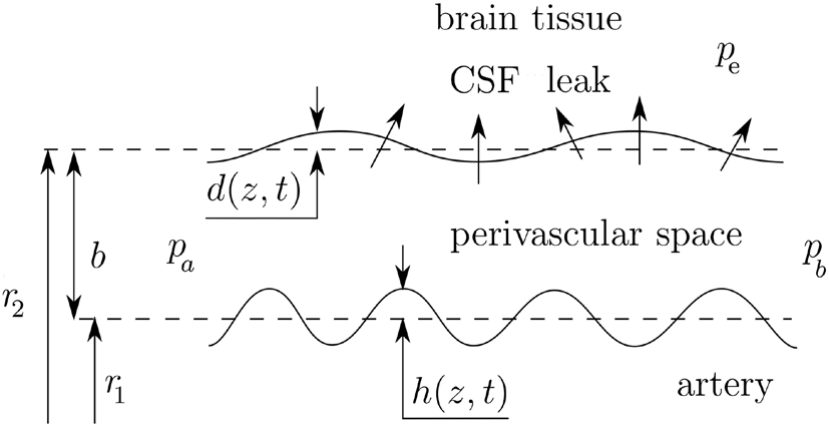
Sketch of the perivascular space between the brain tissue and the artery.

The CSF-filled perivascular space was modeled as a thin annular gap between an elastic, impermeable artery and a brain tissue (Fig. 1). An elastic, permeable glial boundary separates the PVS from the brain tissue. The glial boundary is modeled with a linear elastic wall law and we do not solve for the flow in the artery. Instead, the peristaltic wave deformation of the artery is prescribed as a travelling wave. The interstitial pressure was assumed to be constant and was used as the reference pressure. Linear elastic tube laws were used to relate the deformations of the solid boundaries to the pressure difference across them. Governing equations were simplified using lubrication theory.

The thickness of the PVS is *b*, the average radius of the artery and the average inner radius of the brain tissue are $r_1$ and $r_2$, respectively. The deformation about the average radii are $h(z,t)={\bar{h}}\sin \left[ 2\pi /\lambda \left( z-ct\right) \right]$ and *d*(*z*, *t*), where *h* is the imposed deformation of the travelling peristaltic wave, ${\bar{h}}$, $\lambda$ and *c* its amplitude, wavelength and velocity, respectively, *z* is the traveling (axial) direction, *t* the time, and *d* the glial boundary deformation. The pressure in the brain tissue is $p_{e}$, whereas $p_a$ and $p_b<p_a$ are assumed as pressures at the extrema of the PVS.

### Governing equations

The Navier–Stokes and continuity equations in dimensional form read

1a $$\begin{aligned} \frac{\partial u}{\partial t} + u\frac{\partial u}{\partial r} + w\frac{\partial u}{\partial z}&= -\frac{1}{\rho }\frac{\partial p}{\partial r} + \frac{\mu }{\rho }\left( \frac{1}{r}\frac{\partial u}{\partial r} + \frac{\partial ^2 u}{\partial r^2}+ \frac{\partial ^2 u}{\partial z^2}\right) , \end{aligned}$$

1b $$\begin{aligned} \frac{\partial w}{\partial t} + u\frac{\partial w}{\partial r} + w\frac{\partial w}{\partial z}&= -\frac{1}{\rho }\frac{\partial p}{\partial z} + \frac{\mu }{\rho }\left( \frac{1}{r}\frac{\partial w}{\partial r} + \frac{\partial ^2 w}{\partial r^2}+ \frac{\partial ^2 w}{\partial z^2}\right) , \end{aligned}$$

1c $$\begin{aligned} \frac{1}{r}\frac{\partial (r u)}{\partial r} + \frac{\partial w}{\partial z}&= 0, \end{aligned}$$

where $\vec {u}=(u,w)$ denotes the velocity field in cylindrical coordinates, *p* is the pressure, $\vec {r}=(r,z)$ and *t* are the spatial and temporal coordinates, respectively, $\rho$ is the density of the fluid flowing in the perivascular space, $\mu$ its dynamic viscosity.

Equation (1) are then scaled with

2 $$\begin{aligned} r = b R, \quad z = \lambda Z, \quad t = \frac{T}{\omega }, u = b \omega U, \quad w = \lambda \omega W, \quad p = \frac{\mu \omega }{\varepsilon ^2} P{,} \end{aligned}$$

where $\omega =c/\lambda$ is the frequency of peristaltic wave in the artery, *b* is the thickness of the perivascular fluid film, and $\varepsilon =b/\lambda$. The non-dimensional continuity and Navier–Stokes equations read

3a $$\begin{aligned} \varepsilon ^3\text{ Re }\left( \frac{\partial U}{\partial T} + U\frac{\partial U}{\partial R} + W\frac{\partial U}{\partial Z}\right)&= -\frac{\partial P}{\partial R} + \varepsilon ^2\left( \frac{1}{R}\frac{\partial U}{\partial R} + \frac{\partial ^2 U}{\partial R^2}+ \varepsilon ^2\frac{\partial ^2 U}{\partial Z^2}\right) , \end{aligned}$$

3b $$\begin{aligned} \varepsilon \text{ Re }\left( \frac{\partial W}{\partial T} + U\frac{\partial W}{\partial R} + W\frac{\partial W}{\partial Z}\right)&= -\frac{\partial P}{\partial Z} + \left( \frac{1}{R}\frac{\partial W}{\partial R} + \frac{\partial ^2 W}{\partial R^2}+ \varepsilon ^2\frac{\partial ^2 W}{\partial Z^2}\right) , \end{aligned}$$

3c $$\begin{aligned} \frac{1}{R}\frac{\partial (R U)}{\partial R} + \frac{\partial W}{\partial Z}&= 0, \end{aligned}$$

where $\text{ Re }=\varepsilon \rho \omega b^2/\mu$ is the Reynolds number.

The mathematical problem (3) is closed by the boundary conditions

4a $$\begin{aligned}&Z = 0: P = P_a, \end{aligned}$$

4b $$\begin{aligned}&Z = L: P = P_b, \end{aligned}$$

4c $$\begin{aligned}&R = R_1 + H: U = \partial _T H, \qquad W = 0, \end{aligned}$$

4d $$\begin{aligned}&R = R_1 + 1 + D: \vec {U}\cdot \vec {n} = \partial _T D + M_{e}\left( P-P_{e}\right) , \end{aligned}$$

4e $$\begin{aligned}&{\vec {U}}\cdot {\vec {t}} = 0, \end{aligned}$$

4f $$\begin{aligned}&D = \left( P-P_{e}\right) /E_{e}, \end{aligned}$$

where $M_{e}=k_{g}\mu /b\varepsilon ^2$, $P_{e}$ is the non-dimensional pressure at the glial boundary and $k_{g}$ the permeability of the brain tissue, $E_{e} = E_{g}\varepsilon ^2/\mu \omega (R_1+1)$ and $E_{g}$ the Young’s modulus of the brain tissue. $H=h/b=$ ${\bar{H}}\sin \left[ 2\pi \left( Z-T\right) \right]$ is the imposed deformation of the peristaltic wave of the artery and ${\bar{H}}={\bar{h}}/b$ its amplitude measured from the middle line $R_1=r_1/b$. $D=d/b$ is the deformation of the peristaltic layer at the boundary with the brain tissue; it is measured from the middle line $R_2 = r_2/b = R_1+1$. The normal and tangent unit vector to the glial boundary are denoted by $\vec {n}$ and $\vec {t}$.

### Thin film approximation

Assuming that $\varepsilon \ll 1$, i.e. the wavelenth $\lambda$ of the peristaltic wave is much larger than the film thickness *b*, we expand the pressure, the velocity field and the deformation *D* with the following polynomial series

5a $$\begin{aligned} P&= P_0 + \varepsilon P_1 + \varepsilon ^2 P_2 + \ldots , \end{aligned}$$

5b $$\begin{aligned} U&= U_0 + \varepsilon U_1 + \varepsilon ^2 U_2 + \ldots , \end{aligned}$$

5c $$\begin{aligned} W&= W_0 + \varepsilon W_1 + \varepsilon ^2 W_2 + \ldots , \end{aligned}$$

5d $$\begin{aligned} D&= D_0 + \varepsilon D_1 + \varepsilon ^2 D_2 + \ldots , \end{aligned}$$

where the subscript 0 denotes the solution at the leading order term, ${\mathcal {O}}(\varepsilon ^0)$, 1 refers to the linear correction in $\varepsilon$, ${\mathcal {O}}(\varepsilon ^1)$, 2 indicates the quadratic correction in $\varepsilon$, ${\mathcal {O}}(\varepsilon ^2)$, and so on.

If $\text{ Re }={\mathcal {O}}(1)$ or smaller, the leading order continuity and Navier–Stokes equations read

6a $$\begin{aligned}&\frac{\partial P_0}{\partial R} = 0, \end{aligned}$$

6b $$\begin{aligned}&\frac{\partial P_0}{\partial Z} = \frac{1}{R}\frac{\partial W_0}{\partial R} + \frac{\partial ^2 W_0}{\partial R^2}, \end{aligned}$$

6c $$\begin{aligned}&\frac{1}{R}\frac{\partial (R U_0)}{\partial R} + \frac{\partial W_0}{\partial Z} = 0. \end{aligned}$$

The system (23) is completed by the boundary conditions at leading order

7a $$\begin{aligned}&Z = 0: P_0 = P_a, \end{aligned}$$

7b $$\begin{aligned}&Z = L: P_0 = P_b, \end{aligned}$$

7c $$\begin{aligned}&R = R_1 + H: U_0 = \partial _T H, \qquad W_0 = 0{,} \end{aligned}$$

7d $$\begin{aligned}&R = R_1 + 1 + D_0: {\vec {n}} = (n_r,n_z) = (1, 0), \nonumber \\& {\vec {t}} = (t_r,t_z) = (0, 1), \end{aligned}$$

7e $$\begin{aligned}&U_0 = M_{e}\left( P_0 - P_e\right) + \frac{1}{E_{e}}\frac{\partial \left( P_0 - P_e\right) }{\partial T}, \end{aligned}$$

7f $$\begin{aligned}&W_0 = 0, \end{aligned}$$

7g $$\begin{aligned}&D_0 = \frac{P_0 -P_{e}}{E_{e}}. \end{aligned}$$

More details of the model derivation are given in the section Methods. We however remark that leading order boundary conditions do not include any axial flow at the walls, i.e. $W_0=0$ along the boundaries. For more details about the effect of the axial flow in the glymphatic system, we refer to Albargothy et al.^17^.

The leading order term of the *r*-momentum implies that $P_0$ is only function of *Z* and *T* and, integrating in *r* the *z*-momentum, $W_0$ reads

8 $$\begin{aligned} W_0 = \frac{R^2}{4}\frac{\partial P_0}{\partial Z} + C_1 \ln (R) + C_2, \end{aligned}$$

where $$C_1 = C_1(Z,T)$$ and $$C_2 = C_2(Z,T)$$.

Plugging (8) in the continuity equation and integrating in *R*, we derive the form of $U_0$

9 $$\begin{aligned} U_0 = \frac{C_3}{R} - \frac{R^3}{16}\frac{\partial ^2 P_0}{\partial Z^2} - \frac{R}{4} \left[ 2\ln (R) -1\right] \frac{\partial C_1}{\partial Z} - \frac{R}{2}\frac{\partial C_2}{\partial Z}, \end{aligned}$$

where $$C_3 = C_3(Z,T)$$.

Applying the no-slip boundary conditions at the inner boundary ($R=R_1+H$), i.e. $U_0 = \partial _T H$ and $W_0=0$, and the permeable boundary condition at the outer boundary ($R=R_1+1+D$), i.e. $U_0 = M_{e}(P_0 - P_{e}) + E_{e}^{-1}\partial _T(P_0 - P_{e})$ and $W_0 = 0$, the functions $C_1$, $C_2$ and $C_3$ are expressed in terms of $P_0$ and a second-order differential equation is derived for $P_0$

10 $$\begin{aligned} \frac{\partial P_0}{\partial T} + A_0 \frac{\partial ^2 P_0}{\partial Z^2} + A_1 \frac{\partial P_0}{\partial Z} + A_2 P_{0} = \left[ \frac{E_{e}(R_1+H)}{R_1+1+D_0}\right] \frac{\partial H}{\partial T} + E_{e} M_{e} P_{e} + \frac{\partial P_{e}}{\partial T}. \end{aligned}$$

We refer to the section Methods for the definition of $A_0$, $A_1$, $A_2$. The initial condition for $P_0$ is set to be the linear function consistent with the boundary conditions

11 $$\begin{aligned} P_0(T=0) = P_a(T=0) + \left[ P_b(T=0) - P_a(T=0)\right] Z/L. \end{aligned}$$

Equation (10) is solved numerically by making use of a collocation spectral method in *Z*-direction and discretizing in time by implicit Euler method. The time step employed to discretize in time is always set equal to $\Delta t=10^{-2}$ and 1000 Chebyshev–Gauss–Lobatto nodes are used in *Z*. Further details about the numerical method we chose and its implementation in our solver are reported in the section Methods. In order to avoid non-linearities in the discretization algorithm, the explicit outer-wall deformation $D_0^n$ is employed when computing the solution at time $t_{n+1}$.

We remark that taking into account the recent study by Mestre et al.^4^, the annular space around blood vessels in the brain is not uniform in thickness. In this sense, our axisymmetric approach represents a simplification of the perivascular space geometry assuming that the averaged cross-sectional radius is sufficient to capture the leading-order effects of the CSF dynamics. We stress that such an assumption is at the core of the simplified one-dimensional time-dependent partial differential Eq. (10). Including a non-uniform deformation of the gap cross-section would require a non-trivial extension of the model as the pressure, the glial boundary deformation and the flow velocity should depend by all the three spatial coordinates, i.e. *R*, *Z* and $\Phi$. The resulting system of PDEs would make the extended model far more complex to solve and we expect it would not provide major improvements in the order-of-magnitude estimate of the steady streaming across the glial boundary.

## Results

### Physiological parameters

**Table 1:** Range of the non-dimensional groups for the thin-film problem between an artery and a brain tissue.

Parameter	Description (definition)	Estimated range
$\text{ Re }$	Reynolds number $\left( \varepsilon \rho \omega b^2/\mu \right)$	$[5\times 10^{-12}, \ 2\times 10^{-4}]$
$\varepsilon$	Perivascular film thickness $\left( b/\lambda \right)$	$[10^{-6}, \ 3.75\times 10^{-2}]$
$R_1$	Inner radius of the perivascular layer $\left( r_1/b\right)$	$[0.7, \ 10^3]$
${\bar{H}}$	Amplitude of the peristaltic wave $\left( {\bar{h}}/b\right)$	$[8\times 10^{-4}, \ 0.5]$
*L*	Perivascular length $\left( l/\lambda \right)$	$[2,\ 20]$
$M_{e}$	Permeability of the brain tissue $\left( k_{g} \mu /b \varepsilon ^2\right)$	$[10^{-2},\ 10]$
$E_{e}$	Stiffness of the brain tissue $\left( E_{g}\varepsilon ^2/\mu \omega (R_1+1)\right)$	$[2\times 10^{-10}, \ 0.2]$

Physiologically relevant parameters for the thin liquid film of interest are derived from the literature. Xie et al.^18^ estimates the intracranial pressure to be about $2\times 10^{3}$  Pa, in accordance with Sakka et al.^19^, who report $p_{e}\in [1300 ,\ 2000]$  Pa. Assuming $P_{e}=P_b=0$ as reference pressure in our model, $P_a$ will then be considered in the range $P_a\in [5\times 10^{-7}, 5\times 10^2]$. Wang and Olbricht^8^ report the values measured in previous studies for: the peristaltic wave frequency $\omega \approx 5$  Hz^3^, the inner radius of the perivascular layer $r_1 \approx 10^{-5}$  m^20^, the outer radius of the perivascular layer $r_2 \approx 1.1\times 10^{-5}$  m^21^ (which result in a perivascular film thickness $b \approx 10^{-6}$  m), the peristaltic wave amplitude ${\bar{h}}\in [1.25,\ 5]\times 10^{-7}$  m^22^, the peristaltic wave speed $c\approx 1$  m s$^{-1}$^20,23,24^ (which result in a peristaltic wavelength $\lambda \approx 0.2$  m), and the dynamic viscosity $\mu =9\times 10^{-4}$  Pa s^25^. Plus, the fluid density is comparable to the one of water $\rho \approx \rho _{water} = 10^3$  kg m$^{-3}$. Other values of the artery radius ($r_1 \in [10^{-4}, \ 10^{-3}]$  m) are reported in Thorin-Trescases et al.^26^ Moreover, considering that the artery wall thickness $t_{a}$ is 10 to 100 times smaller than $r_1$, i.e. $t_{a}\in [10^{-6}, \ 10^{-5}]$  m^27^, that the elastic modulus of the artery wall is $E_{a}\in [10^5, \ 10^6]$  N m$^{-2}$ and that the wall density is $\rho _{a}\approx 10^3$  kg m$^{-3}$, the elastic wave speed is $c_{a}=\sqrt{E_{a} t_{a}/2 r_1 \rho _{a}}\in [0.2,\ 7]$  m s$^{-1}$. Considering the relationship given by Atabek^28^, i.e. $c\in [0.1,\ 0.5]c_{a}$, an estimated range of the wave speed of the peristaltic wave can be proposed, which includes the estimate of Wang and Olbrich^8^: $c\in [0.02,3.5]$  m s$^{-1}$ resulting in a range for the peristaltic wavelength $\lambda \in [0.004,\ 0.7]$  m, which includes the previous estimate $\lambda \approx 0.2$  m. Another estimate of *b* is given by Iliff et al.^1^, who reports $b = 10^{-5}$  m, and by Jin et al.^14^, who reports $b = 1.5\times 10^{-4}$  m. Finally, The elastic modulus and the permeability of the brain tissue are $E_{g}\approx 0.01 E_{a}\in [10^3, \ 10^4]$  Pa and $k_{g}\approx 10^{-11}$  m  Pa$^{-1}$  s$^{-1}$, respectively^29,30^. Based on these parameters, the range of the non-dimensional groups of interest for our study is derived and reported in Table 1. From Table 1 it is clear that the leading-order thin-film approach is a very good approximation for our problem since $\varepsilon \ll 1$ and $\text{ Re }\ll 1$. Higher-order corrections in $\varepsilon$ and inertial effects due to $\text{ Re }$ are, therefore, substantially negligible.

### Parametric study

Based on the physiologically relevant parameters, we carried out numerical simulations for the following range of non-dimensional groups

12 $$\begin{aligned} R_1 \in [10,\ 1000],\quad {\bar{H}} \in [0,\ 0.2],\quad L \in [2,\ 20], \quad M_{e} \in [0.1,\ 5],\quad E_{e} \in [0.01,\ 1]. \end{aligned}$$

We remark that the range of the glial boundary elasticity parameter $E_{e}$ has been restricted to vary over 3 rather than 10 orders of magnitude, as indicated in Table 1. Indeed, when the Young modulus of the brain tissue is very small, i.e for the softest brain tissue parameters reported in Table 1, $E_{e}\ll 1$, a small pressure difference across the glial boundary is sufficient to induce significant deformations, hence (7e) reduces to $P_0\approx P_e$ and $U_0\approx 0$. As a result, in the limit $E_e\rightarrow 0$, (10) becomes an instantaneous equation that cannot admit any through flow. For these reasons, we limited our parametric studies to Young moduli related to the most interesting CSF dynamics that can admit steady streaming, i.e. $E_{e} \in [0.01,\ 1]$.

All the simulations are carried out for $t_{fin}=100$ with $\Delta t = 10^{-4}$ and the results are interpreted in terms of brain tissue deformation $D_0$ and through-flow velocity $U_0 - \partial _T D_0$. Since the solution is time-dependent, the corresponding time averages $\langle D_0 \rangle$ and $\langle U_0 - \partial _T D_0 \rangle$ are analyzed, averaging over $t\in [50,100]$ in order to get rid of the initial transient effects. The boundary conditions in pressure are

13 $$\begin{aligned} P_a = 10^{-3}, \qquad P_{e} = 0, \qquad P_{b} = 0. \end{aligned}$$

It is remarkable that, in our model framework, the pressure distribution $P_0(Z,T)$, the deformation of the brain tissue $D_0(Z,T)$ and the through-flow velocity $U_{e}(Z,T) = U_0\vert _{R=R_1+1+D_0} - \partial _T D_0$ can be derived from each other taking into account the permeability parameter $M_{e}$ and the elasticity parameter $E_{e}$

14 $$\begin{aligned} U_{e} = M_{e} P_0, \quad D_0 = E_{e}^{-1}P_0, \end{aligned}$$

hence, analyzing the results in terms of one among $P_0$, $U_{e}$ or $D_0$ provides information about all three these quantities. A direct implication of it is the pressure boundary conditions play the role of boundary constraints for $U_{e}$ and $D_0$, too. Hence, given an elasticity parameter $E_{e}$, regardless of $M_{e}$, ${\bar{H}}$, *L* and $R_1$, the brain tissue deformation at $Z=0$ will always be $D_0\vert _{Z=0} = E_{e}^{-1} P_a$ and at $Z=L$ $D_0\vert _{Z=L} = 0$. With the same argument, fixing $M_{e}$ and regardless of $E_{e}$, ${\bar{H}}$, *L* and $R_1$, the through-flow velocity on the left will always be $U_{e}\vert _{Z=0} = M_{e} P_a$ and on the right $U_{e}\vert _{Z=L} = 0$.

#### Vanishing peristaltic wave

We first consider all the cases with ${\bar{H}}=0$, since the flow reaches a steady state ($\partial _T D_0 = \partial _T P_0=0$) and it can be well understood taking into account the exact solution reported for the flow in an annular pipe with a permeable wall. This represents an asymptotic limit of our problem and can therefore be used as a validation case for our solver. Taking the limit of ${\bar{H}}\rightarrow 0$, $E_{e}\rightarrow \infty$ and assuming $P_{e}$, $P_{a}$ and $P_{b}$ constant in time, the problem admits a steady solution and since $D_0 \equiv 0$, the pressure $P_0$ becomes an instantaneous field (i.e. $\partial _T P_0 \equiv 0$), reducing (10) to

15 $$\begin{aligned} \frac{\partial ^2 P_0}{\partial Z^2} + B P_0 = B P_{e}, \end{aligned}$$

with $A_1\equiv 0$ and the constant *B* given in the section Methods. Considering that *B* is always negative, i.e. $\vert B \vert = -B$, the solution of (15) is of the form

16 $$\begin{aligned} P_0 = P_{e} + \gamma _1 e^{Z \sqrt{\vert B \vert }} + \gamma _2 e^{-Z \sqrt{\vert B \vert }}, \end{aligned}$$

where $\gamma _1$ and $\gamma _2$ are constants determined by applying the boundary conditions at $Z=0$ ($P_0\vert _{Z=0}=P_a$) and $Z=L$ ($P_0\vert _{Z=L}=P_b$)

17a $$\begin{aligned} \gamma _1&= \frac{(P_a-P_{e})e^{- L \sqrt{\vert B\vert }}-\left( P_b - P_{e}\right) }{e^{- L \sqrt{\vert B\vert }} - e^{L \sqrt{\vert B\vert }}}, \quad \gamma _2 = \frac{(P_b-P_{e})-\left( P_a - P_{e}\right) e^{L \sqrt{\vert B\vert }}}{e^{- L \sqrt{\vert B\vert }} - e^{L \sqrt{\vert B\vert }}}. \end{aligned}$$

**Figure 2:**
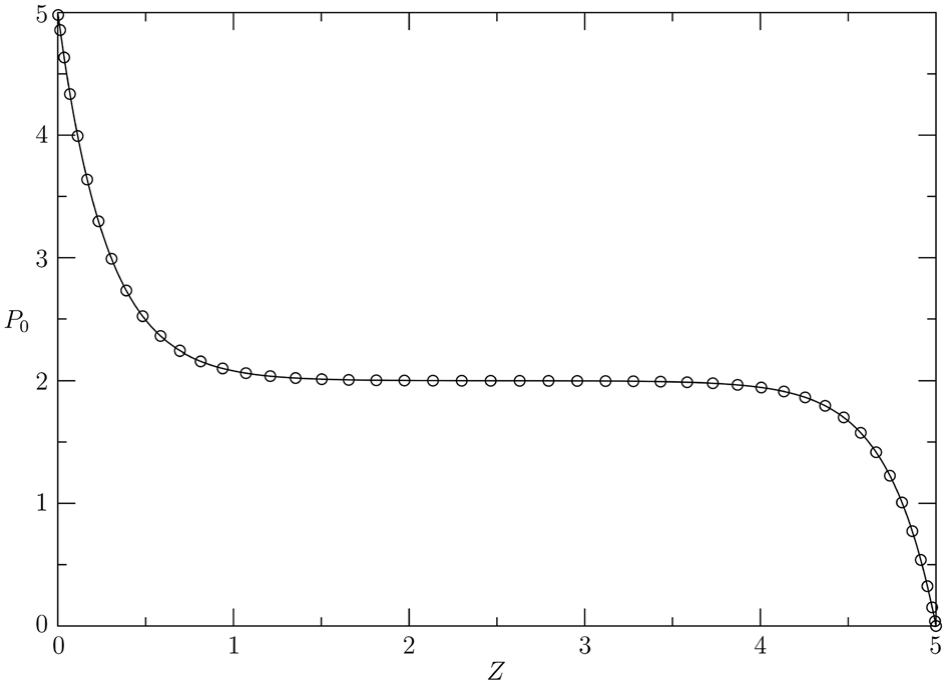
Comparison between the exact steady solution of (16) (solid line) valid for $E_{e}\rightarrow \infty$ and ${\bar{H}}=0$ and the corresponding numerical solution of (10) (circles) evaluated at $t=1$ and computed for ${\bar{H}}=0$ and $E_{e}=10^5$. The other parameters of the comparison are $P_{e}=2$, $P_a=5$, $P_b=0$, $M_{e}=1$, $L=5$ and $R_1=5$.

For validation purpose, the numerical solution of (10) for $E_{e}=10^5$, $P_{e}=2$, $P_a=5$, $P_b=0$, $M_{e}=1$, ${\bar{H}} = 0$, $L=5$ and $R_1=5$ at $t = 1$ is compared with the exact solution (16), valid only for $E_{e}\rightarrow \infty$ and ${\bar{H}} = 0$. The very good agreement is depicted in Fig. 2.

**Figure 3:**
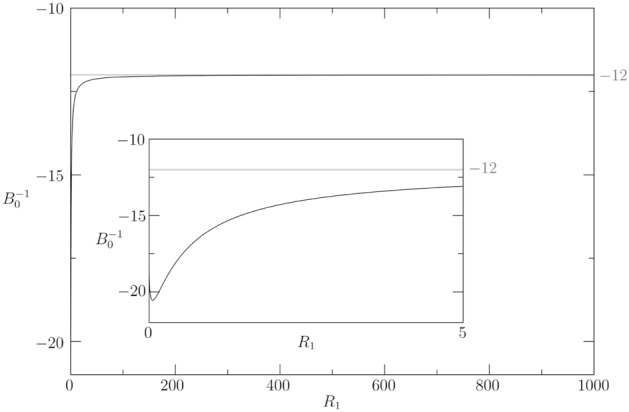
Asymptotic limit of $1/B_0$ to match the plane flow coefficient for $R_1\rightarrow \infty$: $\lim _{R_1\rightarrow \infty } 1/B_0 = -12$.

A further confirmation of the derivation of our model is provided in the limit of large inner radius $R_1$. For $R_1\rightarrow \infty$, the curvature effect becomes negligible and (15) tends to the equation for the incompressible flow in a plane shallow channel with a permeable wall

18 $$\begin{aligned} \frac{\partial ^2 P_0}{\partial X^2} - 12 M_{e} P_0 = - 12 M_{e} P_{e}, \end{aligned}$$

where the non-dimensional plane coordinates are $\vec {X}=(X,Y)$. Equation (18) is derived in the section Methods and it implies that $\lim _{R_1\rightarrow \infty } 1/B_0 = -12$. The correct asymptotic limit of our model is retrieved, as shown in Fig. 3.

**Figure 4:**
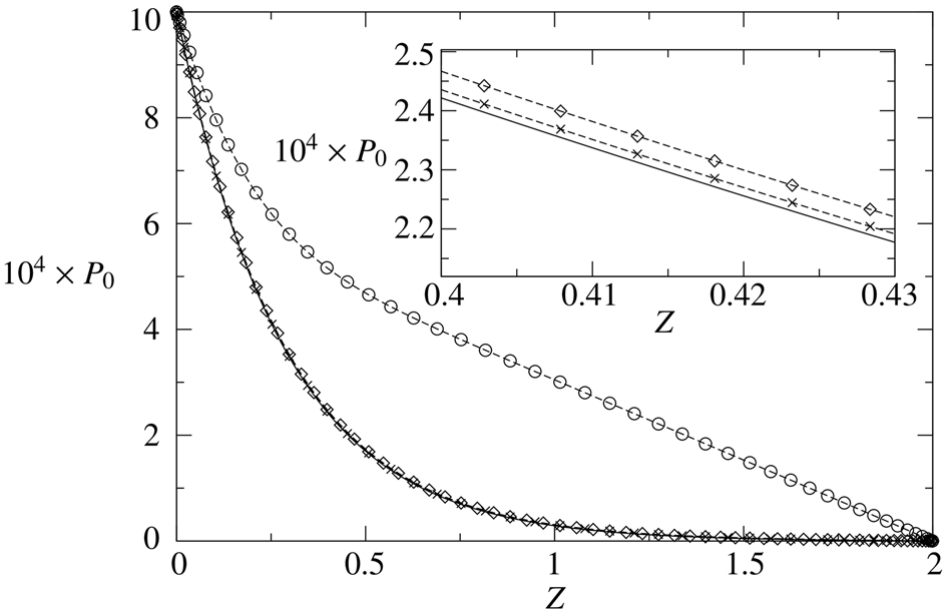
Pressure distribution in a rigid pipe with a permeable wall (solid line) compared to the pressure in the PVS for ${\bar{H}}=0$, $E_{e}=0.01$ (circles and dashed-line), 0.1 (squares and dashed-line) and 1 (crosses and dashed-line). In all the cases $M_{e}=1$, $L=2$, $R_1=10$ (i.e. $B_0=-0.079557$), $P_{e}=P_b=0$ and $P_a= 10^{-3}$ at $t=50$.

Fixing $P_{e}=P_b=0$ and $P_a=10^{-3}$ the pressure in the annular pipe reads:

19 $$\begin{aligned} P_0 = P_a \ \frac{e^{(Z-L)\sqrt{\vert B \vert }} - e^{(L-Z)\sqrt{\vert B \vert }}}{{e^{-L\sqrt{\vert B \vert }}-e^{L\sqrt{\vert B \vert }}}}, \end{aligned}$$

hence, the solution is nothing but an exponential relaxation from $$P_a$$ to $$P_{e}=P_b=0$$. This same trend is observed for all the cases with $${\bar{H}}=0$$, and they are compared in Fig. 4 for $$M_{e}=1$$ and $$E_{e}=0.01$$, 0.1 and 1 at $$t=50$$. Since the annular channel flow is a limit for $$E_{e}\rightarrow \infty $$ (i.e. $$D_0\rightarrow 0$$), upon an increase of stiffness of the brain tissue, the pressure distribution tends to (19). It is remarkable that, for the least rigid brain tissue, i.e. $$E_{e}=0.01$$, the exponential relaxation of $$P_0$$, $$ D_0$$ and $$U_{e}$$ blends soon (i.e. approximately for $$Z>0.5$$) with a linear trend which holds in most of the thin film.

#### Effect of gap length

**Figure 5:**
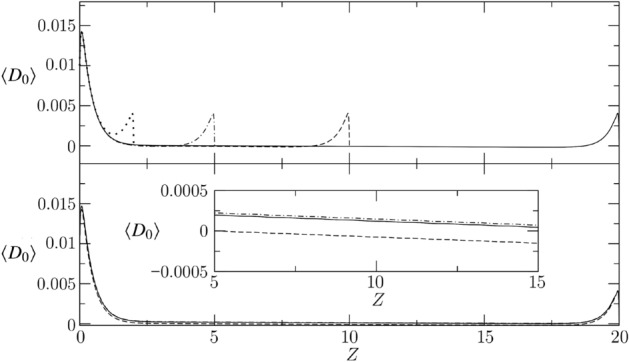
Top: Effect of PVS length for ${\bar{H}} = 0.2$, $R_1 = 10$, $M_{e} = 1$, $E_{e} = 0.1$ is investigated considering four axial lengths: $L=2$ (dotted), $L=5$ (dashed–dotted), $L=10$ (dashed), $L=20$ (solid). Bottom: Effect of curvature for ${\bar{H}} = 0.2$, $L = 20$, $M_{e} = 0.5$, $E_{e} = 0.1$ investigated considering three inner radii: $R_1=10$ (solid line), $R_1=100$ (dashed line) and $R_1=1000$ (dashed–dotted line).

The effect of the gap length *L* is investigated, setting ${\bar{H}} = 0.1$, $R_1 = 10$, $M_{e} = 0.5$, $E_{e} = 0.1$ and varying $L \in [2,\ 20]$. Four PVS lengths are considered and the corresponding average deformation $\langle D_0 \rangle$ is depicted in the top panel of Fig. 5: $L=2$ (dotted), $L=5$ (dashed–dotted), $L=10$ (dashed), $L=20$ (solid). For all the curves it is noticed that the boundary effect which dominates the average deformation distribution is limited to a couple of wavelengths from the boundaries.

The peak at $Z=0$ is well understood considering the equivalence $P_0 = E_{e}^{-1} D_0$, which therefore fixes a steady Dirichlet boundary condition on $D_0(Z=0) = E_{e} P_a$, hence $\langle D_0 \rangle (Z=0) = E_{e} P_a$. This value is much larger than the average deformation in the bulk, since the flow in the bulk is strongly influenced by the permeability of the glial boundary (see Fig. 4). The second peak near $Z=L$ is typical of non-transparent boundary conditions for wave propagation problems; the steep negative gradient of $\langle D_0 \rangle (Z\rightarrow L)$ is a direct results of the Dirichlet boundary condition $D_0(Z=L) = E_{e} P_b = \langle D_0 \rangle (Z = L) = 0$.

In order to get rid of these boundary effects induced by the simplified pressure boundary conditions, we focus on the bulk area where each curve can be well approximated by a straight line, characterized by the only two coefficients $A_0$ and $A_1$

20 $$\begin{aligned} \langle D_0 \rangle \vert _{Z\in [5,L-5]} \approx A_0 + A_1 Z. \end{aligned}$$

where the coefficient $A_0$ represents the time- and space-averaged brain tissue deformation and the coefficient $A_1$ is the time-averaged axial rate of change of the brain tissue deformation. It is further noticed that the average through flow $\langle U_{e} \rangle$ (derived by $\langle D_0 \rangle$ multiplying by $M_{e} E_{e}$) admits a steady streaming since $\int _{5}^{L-5} \langle U_{e} \rangle dZ\ne 0$. The characterization of the steady streaming via $A_0$ and $A_1$ is one of the main aim of our study, as reported in the followings.

#### Effect of curvature

The effect of the curvature is discussed, setting ${\bar{H}} = 0.1$, $L = 20$, $M_{e} = 0.5$, $E_{e} = 0.1$ and varying $R_1 \in [10,\ 1000]$. The bottom panel of Fig. 5 compares the average deformation for the three radii of curvature $R_1=10$ (solid line), $R_1=100$ (dashed line) and $R_1=1000$ (dashed–dotted line). The curvature of the annular PVS has relatively small importance in terms of $\langle D_0 \rangle$. Increasing the curvature ($\downarrow R_1$) does not have a monotonic trend on the average deformation in the middle of the liquid film, and it tends to preserve the peak near the inflow and outflow boundaries. As also confirmed by Fig. 3, which plots the asymptotic limit of $B_0^{-1}$, the curvature effect becomes negligible when comparing $R_1=100$ and $R_1=1000$.

#### Highest curvature ($R_1=10$) and longest perivascular gap ($L=20$)

The following results consider $R_1=10$ and $L=20$. The effect of the peristaltic wave amplitude ${\bar{H}}\in [0,0.2]$ and of the brain tissue permeability $M_{e}\in [0.1,5]$ is investigated for three cases: soft ($E_{e}=0.01$), medium-stiff ($E_{e}=0.1$) and rigid ($E_{e}=1$) brain tissue.

**Figure 6:**
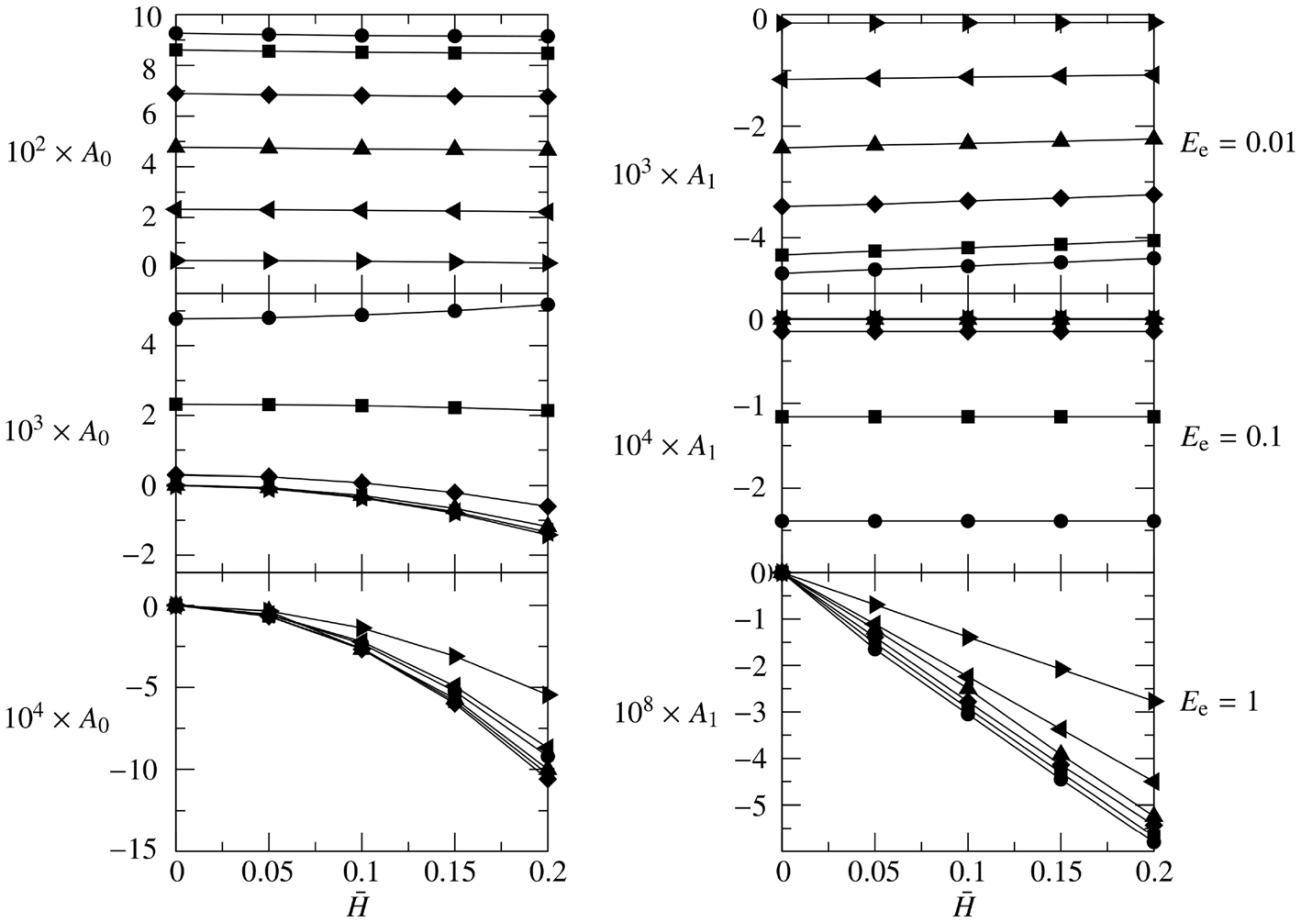
$A_0$ (left panels) and $A_1$ (right panels) coefficients for the average deformation $\langle D_0 \rangle$ for $R_1 = 10$, $L = 20$, $M_{e} \in [0.1,\ 5]$, and $E_{e} = 0.01$ (top), $E_{e} = 0.1$ (middle) and $E_{e} = 1$ (bottom). Six values of $M_{e}$ are considered: $M_{e}=0.1$ ($\bullet$), 0.2 ($\blacksquare$), 0.5 (♦), 1 ($\blacktriangle$), 2 ($\blacktriangleleft$), 5 ($\blacktriangleright$).

##### **Soft brain tissue**

The results of $A_0$ (left-top panel) and $A_1$ (right-top panel) for $E_{e}=0.01$ are depicted in Fig. 6. Five values of ${\bar{H}}$ are considered, i.e. ${\bar{H}}=0$, 0.05, 0.1, 0.15, and 0.2, for each six values of $M_{e}$: $M_{e}=0.1$ ($\bullet$), 0.2 ($\blacksquare$), 0.5 (♦), 1 ($\blacktriangle$), 2 ($\blacktriangleleft$), 5 ($\blacktriangleright$). The case ${\bar{H}}=0$ is the only one admitting a steady state for the flow. This reflects on the time-average deformation $\langle D_0 \rangle$ (and on $\langle P_0 \rangle$ and $\langle U_{e} \rangle$, see (14)), which has an exponential trend matching to a linear profile (see Fig. 4). Moreover, all the cases considered, regardless of $M_{e}$, $E_{e}$ and ${\bar{H}}$, show a decrease of $\langle D_0 \rangle$, $\langle P_0 \rangle$ and $\langle U_{e} \rangle$ as *Z* increases, i.e. $A_1$ is always negative. Upon an increase of ${\bar{H}}$, also the amplitude of the average brain tissue deformation $\langle D_0 \rangle$ increases ($A_1 \uparrow$). The coefficient $A_0$ is monotonically decreasing with ${\bar{H}}$, even if almost negligibly. For soft brain tissues, $A_0$ and $A_1$ strongly depend on the permeability of the brain tissue $M_{e}$. For the parameter ranges investigated, $A_1$ and $A_0$ show a monotonic trend decreasing as $M_{e}$ increases, if $E_{e}=0.01$.

**Figure 7:**
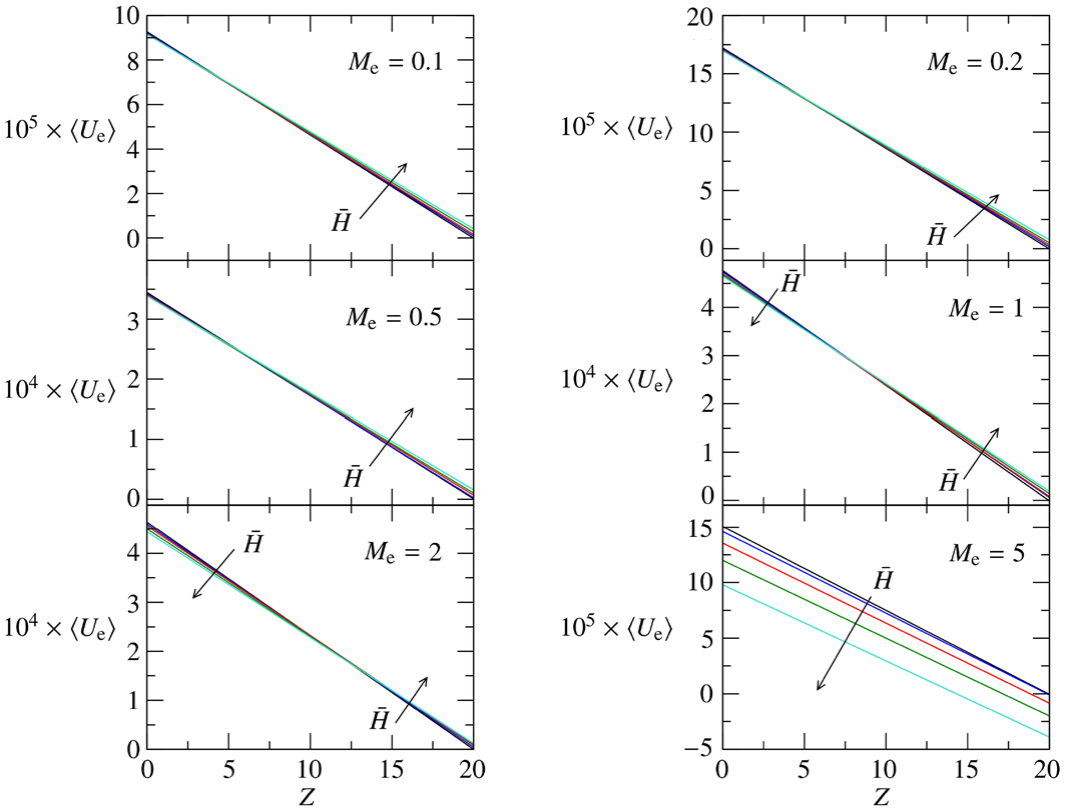
$\langle U_{e} \rangle$ for $R_1 = 10$, $L = 20$, $M_{e} \in [0.1,\ 5]$, and $E_{e} = 0.01$. Five values of ${\bar{H}}$ are considered: ${\bar{H}}=0$ (black), 0.05 (blue), 0.1 (red), 0.15 (green), 0.2 (cyan).

Employing the equivalence $U_{e} = E_{e}M_{e}D_0$, the coefficients $A_0$ and $A_1$ are used to plot the fitting approximation of $\langle U_{e} \rangle$. For soft brain tissues, Fig. 7 reports the time average of the through-flow profile across the glial boundary for $M_{e} \in [0.1,\ 5]$ and ${\bar{H}}\in [0,0.2]$. Each panel of Fig. 7 compares the effect of the peristaltic wave amplitude (black: ${\bar{H}}=0$; blue: ${\bar{H}}=0.05$; red: ${\bar{H}}=0.1$; green: ${\bar{H}}=0.15$; cyan: ${\bar{H}}=0.2$) keeping constant the permeability parameter $M_{e}$ for $E_{e}=0.01$. Figure 7 demonstrates that a steady through flow is due to the peristaltic wave amplitude, which increases the overall through flow, $\int _0^L\langle U_{e} \rangle dZ$, for $M_{e}\le 1$ and decreases it for $M_{e} > 1$.

The understanding of a steady streaming component in the through flow is an interesting outcome of our model. In fact, considering that *H* is a zero-mean deformation, the increase of $\langle P_0 \rangle =E_{e}\langle D_0 \rangle$ with ${\bar{H}}$ highlights the steady pressure component induced by the traveling wave. This is possible only because the brain tissue is deformable $E_{e}\not \rightarrow \infty$, hence $\partial _T D_0 = E_{e}^{-1} \partial _T P_0 \ne 0$. The presence of a time-derivative in (10) allows a phase shift between $D_0$ and *H*. To better understand it, let us consider the case of $E_{e}\rightarrow \infty$ with ${\bar{H}}\ne 0$. Since the lubrication approximation considers only linear terms of the momentum equation, if $E_{e}\rightarrow \infty$, $\partial _T D_0 = E_{e}^{-1} \partial _T P_0 = 0$ and (10) becomes an instantaneous equation. As a consequence, for $E_{e}\rightarrow \infty$, the fluid flow becomes fully reversible in time and a symmetric zero-mean deformation *H*, as the one we consider, would produce a zero-mean streaming $\langle U_{e} \rangle \equiv 0$ within a traveling wave period. For $E_{e}\not \rightarrow \infty$, the time derivative $\partial _T P_0$ carries the memory of the previous states and makes the flow non-reversible in time, which allows for steady streaming.

##### Medium-stiff brain tissue

The results for $E_{e}=0.1$ are depicted in Fig. 6: $A_0$ (left-middle panel) and $A_1$ (right-middle panel). The same line-style coding is used to denote different ${\bar{H}}$, as for the soft tissue case. The first difference with the soft-tissue case is observed in $A_0$, which is one to two orders of magnitude lower than for soft brain tissues. Once again, this is understood considering the steady case (${\bar{H}}=0$), which reduced to an almost-exponential relaxation profile (see squares in Fig. 4). Hence, the linear profile inherited by soft tissues from ${\bar{H}}=0$ vanished for medium-stiff brain tissues reducing $A_0$ of two orders of magnitudes. The increased rigidity $E_e$ further contributes to this reduction of $A_0$ as $D_0 = P_0 E_{e}^{-1}$. Differently from the soft-tissue case, for $E_{e}=0.1$, $A_0$ shows a certain dependence on ${\bar{H}}$, which grows monotonically for small permeability parameters $M_{e}=0.1$ and decreases monotonically when the permeability of the glial boundary is higher. On the other hand, $A_1$ is always negative and independent (up to the accuracy of our numerical simulation) on ${\bar{H}}$, and it is remarkably influenced by $M_{e}$ up to becoming almost zero if the permeability parameter is high enough ($M_{e}\gtrsim 1$). This is well understood considering that a higher $M_{e}$ implies a faster relaxation of the average pressure to a constant value, as indicated by the coefficient *B* of (19). Since $P_0 = U_{e} M_{e}^{-1} = D_0 E_{e}$, this same consideration applies to $\langle D_0 \rangle$ and $\langle U_{e} \rangle$.

**Figure 8:**
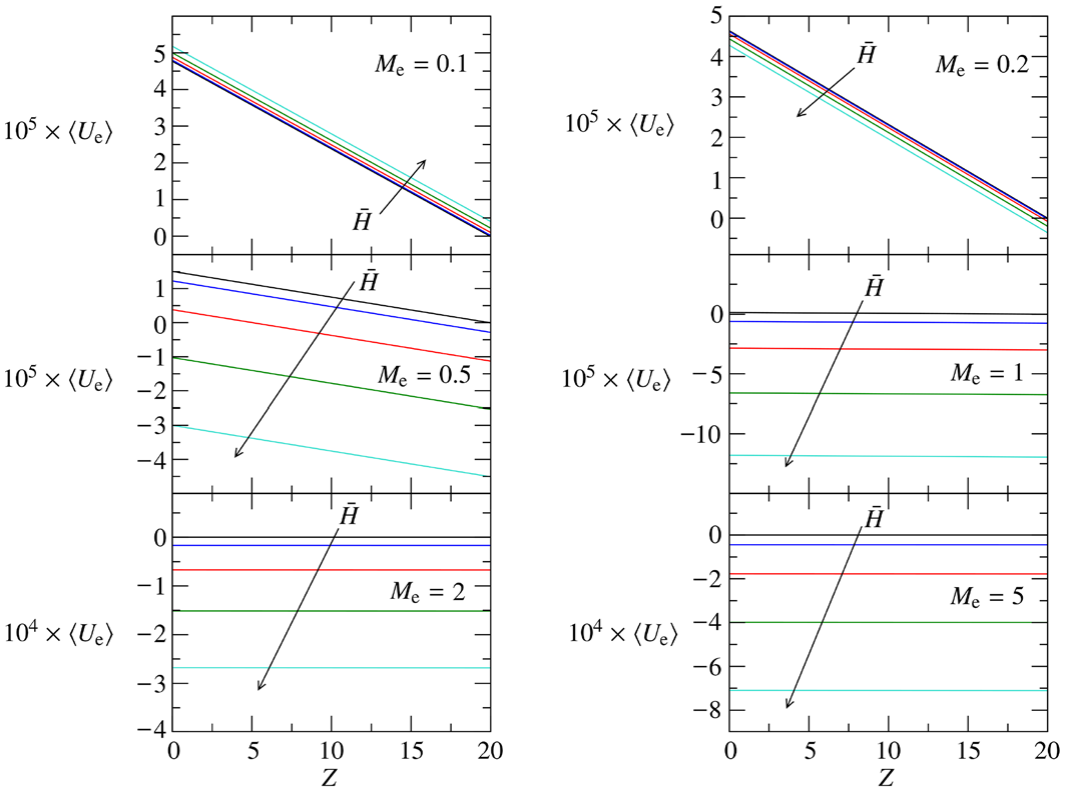
$\langle U_{e} \rangle$ for $R_1 = 10$, $L = 20$, $M_{e} \in [0.1,\ 5]$, and $E_{e} = 0.1$. Five values of ${\bar{H}}$ are considered: ${\bar{H}}=0$ (black), 0.05 (blue), 0.1 (red), 0.15 (green), 0.2 (cyan).

The hallmark of the steady exponential relaxation due to the pressure gradient is hardly visible when comparing the time-dependent profiles of $D_0$ for ${\bar{H}}=0$ and ${\bar{H}}=0.4$. This is the direct consequence of the stiffness parameter, since increasing $E_{e}$ reduces the deformation at $Z=0$ for a given $P_a$, i.e. $D_0\vert _{Z=0} = E_{e}^{-1} P_a$. The flow is then dominated by the peristaltic wave deformation *H* which gives rise to an interesting phenomenon: increasing the permeability parameter $M_{e}$, for very permeable brain tissues $M_{e}\gtrsim 1$, the average brain tissue deformation $\langle D_0 \rangle$ becomes negative.

As a result, using (14), a negative average deformation $\langle D_0 \rangle \ <0$ implies a suction from the brain to the perivascular space $\langle U_{e} \rangle \ < 0$. Hence, increasing $M_{e}$ gives rise to an opposite direction of the steady streaming, which now flows from the brain to the PVS. Based on Fig. 6, the sign change occurs at $M_{e}\approx 0.5$ for ${\bar{H}}\ge 0.15$ and at $M_{e}\approx 1$ $\forall {\bar{H}}$. Figure 8 reports the time average of the through-flow profile for $E_{e}=0.1$, $M_{e} \in [0.1,\ 5]$ and ${\bar{H}}\in [0,0.2]$. The same color coding of Fig. 7 is used. For medium-stiff brain tissues, an increase of the peristaltic wave frequency increases $\langle U_{e} \rangle$ if $M_{e}=0.1$ and decreases $\langle U_{e} \rangle$ if $M_{e}\ge 0.2$, consistently with the trend of $A_0$ for $E_{e}=0.1$.

##### Rigid brain tissue

**Figure 9:**
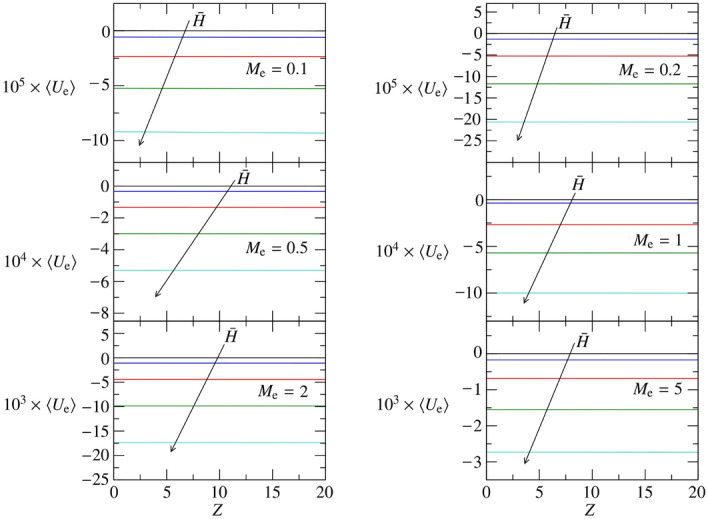
$\langle U_{e} \rangle$ for $R_1 = 10$, $L = 20$, $M_{e} \in [0.1,\ 5]$, and $E_{e} = 1$. Five values of ${\bar{H}}$ are considered: ${\bar{H}}=0$ (black), 0.05 (blue), 0.1 (red), 0.15 (green), 0.2 (cyan).

The results for $E_{e}=1$ are depicted in Fig. 6: $A_0$ (left-bottom panel) and $A_1$ (right-bottom panel) using the same line-style coding of the previous cases. Very similar qualitative considerations done for the medium-stiff brain tissue about $\langle D_0 \rangle$ and $\langle U_{e} \rangle$ apply to the rigid brain tissue. Upon an increase of $E_{e}$, the amplitude of the average deformation $\langle D_0 \rangle$ decreases (as expected, see absolute values of $A_0$ and $A_1$). Indeed, we remark that $\langle D_0 \rangle$ must become steady and converge to zero if the rigidity of the brain tissue goes to infinite, i.e. $\lim _{E_{e}\rightarrow \infty } \langle D_0 \rangle = \lim _{E_{e}\rightarrow \infty } D_0 = 0$. It is furthermore remarkable that, for $E_{e}=1$, the rigidity of the brain tissue further contributes to creating negative deformation regions resulting in $\langle D_0 \rangle$ always negative. This has corresponding implications on $\langle U_{e} \rangle$, which admits more and more extended suction regions, making permeable stiff brain tissues streaming fluid, in average, exclusively from the glial boundary to the perivascular space. This is clearly demonstrated by Fig. 9, where the average through flow across the glial boundary is depicted using the same template of Figs. 7 and 8.

The trend reported in Fig. 6 for $A_0$ has an interesting minimum at about $M_{e}\approx 0.5$. Indeed, the integral balance $A_0\approx \int _0^L D_0 dZ$ becomes smaller and smaller, in absolute value, upon an increase of $M_{e}$, if $M_{e}\ge 0.5$. This behavior is understood considering the competition between two opposite effects: (a) increasing the permeability of the glial boundary, more fluid can go through the brain tissue $\vert \langle U_{e} \rangle \vert \uparrow$ (see Fig. 9), hence increasing $\vert \langle D_0 \rangle \vert$, and (b) increasing the permeability parameter $M_{e}$ the brain tissue will oppose less and less resistance to be penetrated, hence $\vert \langle D_0 \rangle \vert \downarrow$. The first effect is dominant for $M_{e}\le 0.5$, and the absolute value of $A_0$ increases with $M_{e}$; the second effect is more important for $M_{e}\ge 1$. As a result, the deformation of the brain tissue reduces in amplitude more and more, if the permeability parameter $M_{e}\ge 1$, up to asymptotically leading to an undeformed brain tissues, i.e. $\lim _{M_{e}\rightarrow \infty } \langle D_0 \rangle = \lim _{M_{e}\rightarrow \infty } D_0 = 0$. Since $A_1$ is always four orders of magnitude smaller than $A_0$, the linear component of $\langle P_0 \rangle$, $\langle D_0 \rangle$ and $\langle U_{e} \rangle$ can safely be neglected for rigid brain tissues.

## Discussion

The cerebrospinal fluid flow across the glial boundary of the brain tissue has been investigated by means of a tribological model derived from first principles. We demonstrate that the phase shift between the arterial peristaltic wave and the glial boundary deformation is a necessary conditions to break the flow symmetry and have a steady streaming. Depending on the elasticity and permeability parameters of the glial boundary, $E_e$ and $M_e$, the steady streaming either enters or exits the brain. For physiologically relevant parameters, we proved that such flow is almost insensitive to curvature effects of the annular perivascular gap for $R_1>10$, and of the perivascular length if $L>5$. A very comprehensive characterization of the through flow across the glial boundary is provided within our model framework, quantifying the leading order pressure $\langle P_0 \rangle$, deformation $\langle D_0 \rangle$ and through flow $\langle U_{e} \rangle$ across the glial boundary, averaged in time. A reduced order model can be readily derived for such quantities from our model, implementing the fitting functions $\langle P_0 \rangle \approx E_{e}\left( A_0 + A_1 Z\right)$, $\langle D_0 \rangle \approx A_0 + A_1 Z$ and $\langle U_{e} \rangle \approx E_{e}M_{e}\left( A_0 + A_1 Z\right)$ for whatever perivascular space with $R_1>10$ and $L>5$.

Among the major outcomes of our study, we estimate the average leak flow velocity for a large physiologically relevant parameter space, finding that $\langle U_{e} \rangle$ ranges between $-0.0027 \le \langle U_{e} \rangle \le 0.0005$. Considering that typical peristaltic wave frequencies are $\omega \approx 5$  Hz, the dimensional average through flow is between $-0.0135b$  s$^{-1} \le \langle \omega b U_{e} \rangle \le 0.0025b$  s$^{-1}$, where $10^{-6}\le b\le 1.5\times 10^{-4}$  m is the thickness of the perivascular space. Hence, our model estimates that $-2.25$  $\mu$m/s $\le \langle \omega b U_{e} \rangle \le 0.4$  $\mu$m/s. We remark that this result is consistent with experimental measurements and other model results, since $\langle \omega b U_{e} \rangle$ is typically some orders of magnitude smaller than $\max _t \omega b U_{e}$, which is supposed to be in the range of 1 $\mu$m/s $\le \max _t \omega b U_{e} \le 100$ $\mu$m/s. In particular, considering CSF transport in the perivascular space, Faghih and Sharp^15^ also mention that arterial pulsations can account for the physiological flow rates through these high flow-resistant spaces. Overall, our model elucidates the dependence of CSF transport on the factors listed in Table 1, and therefore provides a framework to better understand the effect of physiological parameters on perivascular transport. For example, the model can be used to predict how pathologies known to modify parameters like extracellular matrix stiffness (e.g. glial scarring following central nervous system injury) alter the magnitude and direction of CSF flow. Therefore, in addition to calculating specific flow rates, the model described here improves our conceptual understanding of perivascular transport in the brain.

A few concluding remarks about the model robustness and its possible extensions. Owing to the very small values of the non-dimensional film thickness, i.e. $0.000001<\varepsilon <0.0375$ (see Table 1), the thin-film approximation represents the most insightful and numerically robust leading-order model for Newtonian creeping flows with a permeable boundary. If we consider the complete axisymmetric creeping flow model, the pressure would depend on both coordinates, *R* and *Z*. Still, as $\varepsilon \ll 1$, the pressure would be a very weak function of *R*, and passing from the thin-film to the complete creeping flow model would mean a significant increment of the model complexity with negligible advantages at leading-order. On the other hand, assuming that the *P* does not depend on *R*, as the simplification (6a) does, does not lead to remarkable model inaccuracies. On top of it, owing to the small $\varepsilon$, solving numerically the creeping flow equations is a much more challenging task than solving the thin-film equations because the creeping flow system becomes stiffer and stiffer the smaller $\varepsilon$ is. In a recent paper, Ladron-de-Guevara et al.^31^ point out that a correct modeling of the outflow boundary condition is important when one wants to model perivascular pumping. We further stress that our model does not include any restrictive assumption on the kind of boundary conditions that can be considered. In fact, the extension of the model to pulsatile boundary conditions is straightforwardly achieved by replacing (7a) and (7b) by $Z = 0: P_0 = P_a(t)$ and $Z = L: P_0 = P_b(t)$. We further remark that including the pulsatile nature of the boundary conditions can induce an improvement of the model accuracy, and we propose it as a very relevant objective for future studies.

## Methods

### Analytic details

Plugging the asymptotic expansion (5) into (3), it yields

21a $$\begin{aligned}&\varepsilon ^3\text{ Re }\left[ \frac{\partial U_0}{\partial T} + U_0\frac{\partial U_0}{\partial R} + W_0\frac{\partial U_0}{\partial Z}+ \varepsilon \left( \frac{\partial U_1}{\partial T} + U_1\frac{\partial U_0}{\partial R} + U_0\frac{\partial U_1}{\partial R} + W_1\frac{\partial U_0}{\partial Z} + W_0\frac{\partial U_1}{\partial Z}\right) + {\mathcal {O}}(\varepsilon ^2)\right] \nonumber \\&\quad \quad = -\frac{\partial P_0}{\partial R} -\varepsilon \frac{\partial P_1}{\partial R} + {\mathcal {O}}(\epsilon ^2), \end{aligned}$$

21b $$\begin{aligned}&\varepsilon \text{ Re }\left[ \frac{\partial W_0}{\partial T} + U_0\frac{\partial W_0}{\partial R} + W_0\frac{\partial W_0}{\partial Z}+ \varepsilon \left( \frac{\partial W_1}{\partial T} + U_1\frac{\partial W_0}{\partial R} + U_0\frac{\partial W_1}{\partial R} + W_1\frac{\partial W_0}{\partial Z} + W_0\frac{\partial W_1}{\partial Z}\right) + {\mathcal {O}}(\varepsilon ^2)\right] \nonumber \\&\quad \quad = -\frac{\partial P_0}{\partial Z} -\varepsilon \frac{\partial P_1}{\partial Z} + \frac{1}{R}\frac{\partial W_0}{\partial R} + \frac{\varepsilon }{R}\frac{\partial W_1}{\partial R} + \frac{\partial ^2 W_0}{\partial R^2} + \varepsilon \frac{\partial ^2 W_1}{\partial R^2} + {\mathcal {O}}(\varepsilon ^2), \end{aligned}$$

21c $$\begin{aligned}&\frac{1}{R}\left[ \frac{\partial (R U_0)}{\partial R} + \varepsilon \frac{\partial (R U_1)}{\partial R}\right] + \frac{\partial W_0}{\partial Z} + \varepsilon \frac{\partial W_1}{\partial Z} +{\mathcal {O}}(\varepsilon ^2) = 0. \end{aligned}$$

Expanding the boundary conditions (4) leads

22a $$\begin{aligned} Z = 0:&\quad P_0 + \varepsilon P_1 + {\mathcal {O}}(\varepsilon ^2) = P_a{,} \end{aligned}$$

22b $$\begin{aligned} Z = L:&\quad P_0 + \varepsilon P_1 + {\mathcal {O}}(\varepsilon ^2) = P_b{,} \end{aligned}$$

22c $$\begin{aligned} R = R_1 + H:&\quad U_0 + \varepsilon U_1 + {\mathcal {O}}(\varepsilon ^2) = \partial _T H, \end{aligned}$$

22d $$\begin{aligned}& W_0 + \varepsilon W_1 + {\mathcal {O}}(\varepsilon ^2) = 0{,} \end{aligned}$$

22e $$\begin{aligned} R = R_1 + 1 + D:&\quad \vec {n} = (n_z,n_r) = \frac{\left[ -\varepsilon \partial _Z D_0 + {\mathcal {O}}(\varepsilon ^2), 1\right] }{\sqrt{1+\varepsilon ^2 \left( \partial _Z D_0\right) ^2 + {\mathcal {O}}(\varepsilon ^3)}}{,} \nonumber \\&\quad \vec {t} = (t_z,t_r) = \frac{\left[ 1, \varepsilon \partial _Z D_0 + {\mathcal {O}}(\varepsilon ^2)\right] }{\sqrt{1+\varepsilon ^2 \left( \partial _Z D_0\right) ^2 + {\mathcal {O}}(\varepsilon ^3)}}{,} \nonumber \\&\quad \frac{U_0 + \varepsilon U_1 -\varepsilon \partial _Z D_0 W_0 + {\mathcal {O}}(\varepsilon ^2)}{\sqrt{1+\varepsilon ^2 \left( \partial _Z D_0\right) ^2 + {\mathcal {O}}(\varepsilon ^3)}} = M_{e}\left( P_0 + \varepsilon P_1 - P_e + {\mathcal {O}}(\varepsilon ^2)\right) \nonumber \\&\quad \quad \quad \quad + \partial _T \left( P_0 + \varepsilon P_1 - P_e + {\mathcal {O}}(\varepsilon ^2)\right) /E_{e}{,} \nonumber \\&\quad W_0 + \varepsilon W_1 + \varepsilon U_0 \partial _Z D_0 + {\mathcal {O}}(\varepsilon ^2) = 0{,} \nonumber \\&\quad D_0 + \varepsilon D_1 + {\mathcal {O}}(\varepsilon ^2) = \left( P_0 + \varepsilon P_1 + {\mathcal {O}}(\varepsilon ^2)-P_{e}\right) /E_{e}, \end{aligned}$$

If $\text{ Re }={\mathcal {O}}(1)$ or smaller, the leading order continuity and Navier–Stokes equation read

23a $$\begin{aligned}&\frac{\partial P_0}{\partial R} = 0, \end{aligned}$$

23b $$\begin{aligned}&\frac{\partial P_0}{\partial Z} = \frac{1}{R}\frac{\partial W_0}{\partial R} + \frac{\partial ^2 W_0}{\partial R^2}, \end{aligned}$$

23c $$\begin{aligned}&\frac{1}{R}\frac{\partial (R U_0)}{\partial R} + \frac{\partial W_0}{\partial Z} = 0. \end{aligned}$$

The system (23) is completed by the boundary conditions at leading order

24a $$\begin{aligned} Z = 0:&\quad P_0 = P_a, \end{aligned}$$

24b $$\begin{aligned} Z = L:&\quad P_0 = P_b, \end{aligned}$$

24c $$\begin{aligned} R = R_1 + H:&\quad U_0 = \partial _T H, \qquad W_0 = 0{,} \end{aligned}$$

24d $$\begin{aligned} R = R_1 + 1 + D:&\quad \vec {n} = (n_z,n_r) = (0, 1), \nonumber \\&\quad \vec {t} = (t_z,t_r) = (1, 0), \end{aligned}$$

24e $$\begin{aligned}&\quad U_0 = M_{e}\left( P_0 - P_e\right) + \frac{1}{E_{e}}\frac{\partial \left( P_0 - P_e\right) }{\partial T}, \end{aligned}$$

24f $$\begin{aligned}&\quad W_0 = 0, \end{aligned}$$

24g $$\begin{aligned}&\quad D_0 = \frac{P_0 -P_{e}}{E_{e}}. \end{aligned}$$

Equation (23)b can be recast in the form

25 $$\begin{aligned} R\frac{\partial P_0}{\partial Z} = \frac{\partial }{\partial R}\left( R\frac{\partial W_0}{\partial R}\right) , \end{aligned}$$

keeping in mind that $P_0$ is just function of *Z* and *T*, and integrating in *R*, it yields

26 $$\begin{aligned} \frac{R^2}{2}\frac{\partial P_0}{\partial Z} + C_1= R\frac{\partial W_0}{\partial R}, \end{aligned}$$

where $C_1$ is just function of *Z* and *T*. Dividing by *R* and integrating once again in radial direction, it yields

27 $$\begin{aligned} \frac{R^2}{4}\frac{\partial P_0}{\partial Z} + C_1 \ln (R) + C_2 = W_0, \end{aligned}$$

which corresponds to (8), where $C_2$ is just function of *Z* and *T*. The leading-order boundary conditions in $W_0$ are $W_0\vert _{R = R_1 + H} = 0$ and $W_0\vert _{R = R_1 + 1 + D_0} = 0$. Substituting them in (27) yields

28a $$\begin{aligned} W_0\vert _{R = R_1 + H}&= \frac{\left( R_1+H\right) ^2}{4}\frac{\partial P_0}{\partial Z} + C_1 \ln \left( R_1+H\right) + C_2 = 0, \end{aligned}$$

28b $$\begin{aligned} W_0\vert _{R = R_1 + 1 + D_0}&= \frac{\left( R_1 + 1 + D_0\right) ^2}{4}\frac{\partial P_0}{\partial Z} + C_1 \ln \left( R_1 + 1 + D_0\right) + C_2 = 0. \end{aligned}$$

Subtracting the two equations, we eliminate $C_2$, and determine $C_1$

29 $$\begin{aligned} C_1 = \alpha \frac{\partial P_0}{\partial Z}, \qquad \alpha = \frac{(R_1+H)^2-(R_1+1+D_0)^2}{4\left[ \ln (R_1+1+D_0) - \ln (R_1+H)\right] }. \end{aligned}$$

By substitution of $C_1$ in (28a), $C_2$ is determined

30 $$\begin{aligned} C_2 = \beta \frac{\partial P_0}{\partial Z}, \qquad \beta = -\frac{(R_1+H)^2}{4} - \alpha \ln (R_1+H), \end{aligned}$$

where $\alpha$ and $\beta$ are functions of *Z* and *T*.

Equation (23)c can be recast in the form

31 $$\begin{aligned} \frac{\partial \left( R U_0\right) }{\partial R} = -R \frac{\partial W_0}{\partial Z}. \end{aligned}$$

Substituting (27) into (31), it reads

32 $$\begin{aligned} \frac{\partial \left( R U_0\right) }{\partial R} = -\frac{R^3}{4} \frac{\partial ^2 P_0}{\partial Z^2} -R\ln (R) \frac{\partial C_1}{\partial Z} -R \frac{\partial C_2}{\partial Z}, \end{aligned}$$

and integrating yields

33 $$\begin{aligned} R U_0 = C_3 -\frac{R^4}{16} \frac{\partial ^2 P_0}{\partial Z^2} - \frac{R^2\left[ 2\ln (R) - 1\right] }{4} \frac{\partial C_1}{\partial Z} - \frac{R^2}{2} \frac{\partial C_2}{\partial Z}, \end{aligned}$$

where $C_3$ is a function of *Z* and *T*. Dividing by *R*, (9) is retrieved

34 $$\begin{aligned} U_0 = \frac{C_3}{R} -\frac{R^3}{16} \frac{\partial ^2 P_0}{\partial Z^2} - \frac{R\left[ 2\ln (R) - 1\right] }{4} \frac{\partial C_1}{\partial Z} - \frac{R}{2} \frac{\partial C_2}{\partial Z}. \end{aligned}$$

Applying the leading-order boundary conditions on $U_0$, yields

35a $$\begin{aligned} U_0\vert _{R = R_1 + H}&= \frac{C_3}{R_1 + H} -\frac{(R_1 + H)^3}{16} \frac{\partial ^2 P_0}{\partial Z^2} - \frac{(R_1 + H)\left[ 2\ln (R_1 + H) - 1\right] }{4} \frac{\partial C_1}{\partial Z} - \frac{(R_1 + H)}{2} \frac{\partial C_2}{\partial Z} = \frac{\partial H}{\partial T}, \end{aligned}$$

35b $$\begin{aligned} U_0\vert _{R = R_1 + 1 + D_0}&= \frac{C_3}{R_1 + 1 + D_0} -\frac{(R_1 + 1 + D_0)^3}{16} \frac{\partial ^2 P_0}{\partial Z^2} - \frac{(R_1 + 1 + D_0)\left[ 2\ln (R_1 + 1 + D_0) - 1\right] }{4} \frac{\partial C_1}{\partial Z} - \frac{(R_1 + 1 + D_0)}{2} \frac{\partial C_2}{\partial Z} \nonumber \\&= M_{e}\left( P_0 - P_{e}\right) +\frac{\partial D_0}{\partial T} = M_{e}\left( P_0 - P_{e}\right) + \frac{1}{E_{e}}\frac{\partial \left( P_0 - P_{e}\right) }{\partial T}. \end{aligned}$$

Eliminating $C_3$ by combining (35a) and (35b) leads to

36 $$\begin{aligned}&\left( R_1+H\right) \left\{ \frac{\partial H}{\partial T} + \frac{(R_1 + H)^3}{16} \frac{\partial ^2 P_0}{\partial Z^2} + \frac{(R_1 + H)\left[ 2\ln (R_1 + H) - 1\right] }{4} \frac{\partial C_1}{\partial Z} + \frac{(R_1 + H)}{2} \frac{\partial C_2}{\partial Z} \right\} =\left( R_1 + 1 + D_0\right) \left\{ M_{e}\left( P_0 - P_{e}\right) \right. \nonumber \\&\left. + \frac{1}{E_{e}}\frac{\partial \left( P_0-P_{e}\right) }{\partial T} + \frac{(R_1 + 1 + D_0)^3}{16} \frac{\partial ^2 P_0}{\partial Z^2} + \frac{(R_1 + 1 + D_0)}{2} \frac{\partial C_2}{\partial Z} + \frac{(R_1 + 1 + D_0)\left[ 2\ln (R_1 + 1 + D_0) - 1\right] }{4} \frac{\partial C_1}{\partial Z} \right\} , \end{aligned}$$

which is equivalent to (10). The coefficient $C_3$ is then computed by substituting the solution $P_0$ and its derivatives in (35a). The coefficients in (10) are

37a $$\begin{aligned} A_0&= \frac{E_{e}\alpha }{4}\left\{ \left( R_1+1+D_0\right) \left[ 2\ln \left( R_1+1+D_0\right) -1\right] - \frac{(R_1+H)^2}{R_1+1+D_0}\left[ 2\ln (R_1+H)-1\right] \right\} \nonumber \\&+ \frac{E_{e}\beta }{2}\left[ \left( R_1+1+D_0\right) -\frac{(R_1+H)^2}{\left( R_1+1+D_0\right) }\right] + \frac{E_{e}}{16}\left[ \left( R_1+1+D_0\right) ^3-\frac{(R_1+H)^4}{R_1+1+D_0}\right] , \end{aligned}$$

37b $$\begin{aligned} A_1&= \frac{E_{e}}{4}\frac{\partial \alpha }{\partial Z}\left\{ \left( R_1+1+D_0\right) \left[ 2\ln \left( R_1+1+D_0\right) -1\right] - \frac{(R_1+H)^2}{R_1+1+D_0}\left[ 2\ln (R_1+H)-1\right] \right\} \nonumber \\&\ \ + \frac{E_{e}}{2}\frac{\partial \beta }{\partial Z}\left[ \left( R_1+1+D_0\right) -\frac{(R_1+H)^2}{\left( R_1+1+D_0\right) }\right] , \end{aligned}$$

37c $$\begin{aligned} A_2&= E_{e} M_{e}, \end{aligned}$$

37d $$\begin{aligned} \alpha&= \frac{(R_1+H)^2-(R_1+1+D_0)^2}{4\left[ \ln (R_1+1+D_0) - \ln (R_1+H)\right] }{,} \end{aligned}$$

37e $$\begin{aligned} \beta&= -\frac{(R_1+H)^2}{4} - \alpha \ln (R_1+H), \end{aligned}$$

37f $$\begin{aligned} C_1&= \alpha \frac{\partial P_0}{\partial Z}, \end{aligned}$$

37g $$\begin{aligned} C_2&= \beta \frac{\partial P_0}{\partial Z}, \end{aligned}$$

37h $$\begin{aligned} C_3&= (R_1+H)\left\{ \frac{\partial H}{\partial T}+\left[ \frac{(R_1+H)^3}{16}\right] \frac{\partial ^2 P_0}{\partial Z^2} + \frac{R_1+H}{4}\left[ 2\ln (R_1+H)-1\right] \frac{\partial C_1}{\partial Z} + \left( \frac{R_1+H}{2}\right) \frac{\partial C_2}{\partial Z}\right\} . \end{aligned}$$

The coefficient *B* of (15) is defined by

38a $$\begin{aligned} B_0&= \frac{A_0\vert _{H=0, \ D_0 = 0}}{E_{e}} = \frac{\alpha \vert _{H=0, \ D_0 = 0}}{4}\left\{ \left( R_1+1\right) \left[ 2\ln \left( R_1+1\right) -1\right] - \frac{R_1^2}{R_1+1}\left[ 2\ln (R_1)-1\right] \right\} \nonumber \\&\ \ + \frac{\beta \vert _{H=0, \ D_0 = 0}}{2}\left[ \left( R_1+1\right) -\frac{R_1^2}{R_1+1}\right] + \frac{1}{16}\left[ \left( R_1+1\right) ^3-\frac{R_1^4}{R_1+1}\right] , \end{aligned}$$

38b $$\begin{aligned} B_2&= \frac{A_2\vert _{H=0, \ D_0 = 0}}{E_{e}} = M_{e}, \end{aligned}$$

38c $$\begin{aligned} \alpha \vert _{H=0, \ D_0 = 0}&= \frac{R_1^2-(R_1+1)^2}{4\left[ \ln (R_1+1) - \ln (R_1)\right] }{,} \end{aligned}$$

38d $$\begin{aligned} \beta \vert _{H=0, \ D_0 = 0}&= -\frac{R_1^2}{4} - \alpha \ln (R_1), \end{aligned}$$

38e $$\begin{aligned} B&= \frac{B_2}{B_0}. \end{aligned}$$

### Shallow channel with a permeable wall

If $E_{e}\rightarrow \infty$, ${\bar{H}}=0$ and $R_1\rightarrow \infty$, the flow in a shallow channel with a permeable wall represents an asymptotic limit of our thin film problem. Denoting the channel height with *b* and the channel length with *L*, if $L\gg b$, and using the scaling (2), the non-dimensional channel flow problem at leading order reads

39a $$\begin{aligned} \frac{\partial P_0}{\partial Y}&= 0, \end{aligned}$$

39b $$\begin{aligned} \frac{\partial P_0}{\partial X}&= \frac{\partial ^2 U_0}{\partial Y^2}, \end{aligned}$$

39c $$\begin{aligned} \frac{\partial U_0}{\partial X} + \frac{\partial V_0}{\partial Y}&= 0. \end{aligned}$$

where $\vec {X}=(X,Y)$ denotes the non-dimensional plane coordinates, $P_0$ and $\vec {U}_0=(U_0,V_0)$ are the pressure and the velocity field at leading order. The system of Eqs. (39) is completed by the boundary conditions

40a $$\begin{aligned} V_0\vert _{Y=0}&= 0, \end{aligned}$$

40b $$\begin{aligned} V_0\vert _{Y=1}&=M_{e} (P_0 - P_{e}), \end{aligned}$$

40c $$\begin{aligned} U_0\vert _{Y=0}&= 0, \end{aligned}$$

40d $$\begin{aligned} U_0\vert _{Y=1}&= 0, \end{aligned}$$

40e $$\begin{aligned} P_0\vert _{X=0}&= P_a, \end{aligned}$$

40f $$\begin{aligned} P_0\vert _{X=L}&= P_b. \end{aligned}$$

Considering that $P_0$ is only function of *X*, and integrating the *X*-momentum twice in *Y*, it yields

41 $$\begin{aligned} U_0 = \frac{\partial P_0}{\partial X}\frac{Y^2}{2} + {\tilde{C}}_1 Y + {\tilde{C}}_2. \end{aligned}$$

Applying the boundary conditions in *Y* direction, we find ${\tilde{C}}_1=-1/2 \ \partial _X P_0$ and ${\tilde{C}}_2=0$. Plugging $\partial _X U_0$ in the continuity equation and integrating once in *Y*-direction, it yields

42 $$\begin{aligned} V_0 = -\frac{1}{2}\frac{\partial ^2 P_0}{\partial X^2}\left( \frac{Y^3}{3} -\frac{Y^2}{2}\right) + {\tilde{C}}_3. \end{aligned}$$

Applying the boundary conditions in *Y* direction, we find ${\tilde{C}}_3=0$ and the following relation for $P_0$ holds

43 $$\begin{aligned} \frac{\partial ^2 P_0}{\partial X^2} - 12 M_{e} P_0 = -12 M_{e} P_{e}. \end{aligned}$$

### Convergence test

The Navier–Stokes and continuity equation of an incompressible flow in a perivascular thin film have been reduced to the solution of an equation in the form

44 $$\begin{aligned} \frac{\partial f}{\partial t} + \alpha \frac{\partial f}{\partial s^2} + \beta \frac{\partial f}{\partial s} + \gamma f = \sigma , \end{aligned}$$

where $\alpha = \alpha (s,t)$, $\beta = \beta (s,t)$, $\gamma = \gamma (s,t)$ and $\sigma = \sigma (s,t)$ are known functions, $s\in [0,\Lambda ]$ is the space variable (*Z* in our thin-film flow) and $t\in [0,t_{fin}]$ denotes the time variable.

We discretize (44) in space making use of a spectral collocation method which employs Gauss–Lobatto nodes based on Chebyshev polynomials. Denoting $[D_N]\in {\mathbb {R}}^{N\times N}$ and $[D_N^2]\in {\mathbb {R}}^{N\times N}$ the first- and second-order discrete derivation matrices in space constructed using *N* Chebyshev–Gauss–Lobatto nodes, (44) discretized in *s* reads

45 $$\begin{aligned} \frac{\partial \vec {f}_N}{\partial t} + \vec {\alpha }_N \left( [D_N^2] \vec {f}_N\right) + \vec {\beta }_N \left( [D_N] \vec {f}_N\right) + \vec {\gamma }_N \vec {f}_N = \vec {\sigma }_N, \end{aligned}$$

where $\vec {f}_N$, $\vec {\alpha }_N$, $\vec {\beta }_N$, $\vec {\gamma }_N$ and $\vec {\sigma }_N$ are $N\times 1$ arrays which gather the values of *f*, $\alpha$, $\beta$, $\gamma$ and $\sigma$ at the location of the *N* nodes at each instant of time *t*. The time discretization is carried out using the implicit Euler scheme. Denoting with $t_n$ the current time and with $t_{n+1}$ the next instant such that $\Delta t = t_{n+1}-t_n$, the time-discrete version of (45) reads

46 $$\begin{aligned}&\left\{ [I_N]/\Delta t + \text {diag}\left( \vec {\alpha }_N^{n+1}\right) [D_N^2] + \text {diag}\left( \vec {\beta }_N^{n+1}\right) [D_N] + \text {diag}\left( \vec {\gamma }_N^{n+1}\right) \right\} \vec {f}_N^{n+1} = \vec {\sigma }^{n+1}_N + \vec {f}_N^{n}/\Delta t, \end{aligned}$$

where the superscripts *n* and $n+1$ denote the times $t_n$ and $t_{n+1}$, respectively, $[I_N]$ is the $N\times N$ identity matrix and $\text {diag}\left( *\right)$ is the diagonal matrix resulting from distributing the $N\times 1$ array $*$ along the diagonal of an $N\times N$ matrix.

**Figure 10:**
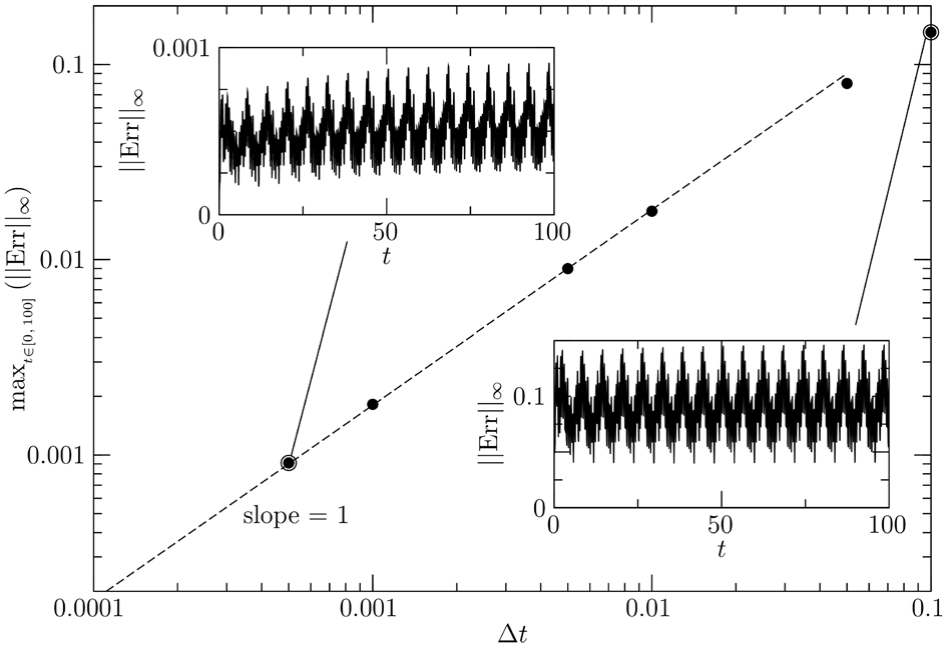
Maximum in time of the infinite norm of the error function ($\vert \vert \text {Err}\vert \vert _\infty$, bullets), slope-1 line assumed as reference for the solver accuracy (dashed line). The two insets depict the infinite norm of the numerical error $\vert \vert \text {Err}\vert \vert _\infty$ as function of *t* for $\Delta t = 0.1$ and 0.0005.

To test the numerical implementation of our code, we assume

47a $$\begin{aligned} \alpha&= -\sin \left[ 2\pi (s-t)\right] - 1.05, \end{aligned}$$

47b $$\begin{aligned} \beta&= \sin \left[ \pi (s-2t)/6\right] , \end{aligned}$$

47c $$\begin{aligned} \gamma&= \cos (2s), \end{aligned}$$

47d $$\begin{aligned} \sigma&= \pi \cos \left[ \pi (s+t)\right] - \alpha \pi ^2\sin \left[ \pi (s+t)\right] + \beta \pi \cos \left[ \pi (s+t)\right] + \gamma \sin \left[ \pi (s+t)\right] , \end{aligned}$$

such that the exact solution of (44) is

48 $$\begin{aligned} f(s,t) = \sin \left[ \pi (s+t)\right] . \end{aligned}$$

Dirichlet boundary conditions are derived from (48) and set at $s=0$ and $s=\Lambda =4$ in (46), together with the initial condition $\vec {f}_N^0=f(\vec {s}_N,t=0)=\sin \left( \pi \vec {s}_N\right)$. We stress that the arbitrary choices made in (47) are representative of the problem of interest in our study.

The numerical solution $\vec {f}_N$ is then compared to the exact solution at each time step by means of the infinite norm of the error function $\text {Err}(t^n) = f(\vec {s}_N,t=t^n) - \vec {f}_N^n$ computed at each time point. The simulations are carried out for $t=t_{fin}=100$ setting $N=100$ and varying $\Delta t$. Figure 10 depicts the convergence curve of the error function, which demonstrate the correctness of our numerical code. The bullets denote the maximum in time of $\vert \vert \text {Err}\vert \vert _\infty$, depicting it in a log-log plot against the $\Delta t$ to demonstrate that the solver is first-order accurate in time (see dashed line with slope 1), as expected. The infinite norm of the numerical error $\vert \vert \text {Err}\vert \vert _\infty$ is plotted as function of time for the largest and the smallest time step (i.e. $\Delta t = 0.1$ and 0.0005, respectively) in the two insets of fig. 10.

## Data Availability

The code used in this paper is an in-house developed software that will be made available upon request to the corresponding author.
